# Management Strategies for Adverse Events Associated With EGFR TKIs in Non–Small Cell Lung Cancer

**Published:** 2016-11-01

**Authors:** Wendy H. Vogel, Paul Jennifer

**Affiliations:** Wellmont Cancer Institute, Kingsport, Tennessee, and University of North Carolina, Chapel HIll, North Carolina

## Abstract

New advances in the treatment of non–small cell lung cancer (NSCLC) have afforded patients longer progression-free survival times, but these therapies are also associated with specific side effects that may not be seen with chemotherapy or radiotherapy. One class of agents includes the epidermal growth factor receptor (EGFR) tyrosine kinase inhibitors (TKIs), which have been shown to be efficacious in patients whose tumors harbor *EGFR*-activating mutations. Certain adverse effects, particularly rash and diarrhea, as well as mucositis/stomatitis, paronychia, ocular disorders, and interstitial lung disease, are seen with this class as a function of their mechanism of action. This review presents the suggested pathogenesis of these toxicities as well as specific management strategies to assist advanced practitioners in helping patients receive the full benefit of treatment with EGFR TKIs.

Lung cancer is the second most common cancer and the leading cause of cancer-related death in the United States, with approximately 221,200 new cases and 158,040 deaths estimated in 2015 (Siegel, Miller, & Jemal, 2015). Non–small cell lung cancer (NSCLC) accounts for about 85% of all lung cancer cases, and most patients are diagnosed at an advanced stage (stage III or IV), often when surgical resection is no longer a viable option (National Cancer Institute [NCI], 2015a; Pilkington et al., 2015).

Traditional treatment modalities for patients with advanced lung disease are chemotherapy, radiation therapy, bevacizumab (Avastin, a targeted agent against vascular endothelial growth factor [VEGF]), or a combination of these options; however, despite multimodal treatment, high rates of local and distant failure are common, and adverse events can be serious (Ramnath et al., 2013; Huber, Reck, & Thomas, 2013; Pilkington et al., 2015; Sun, Ma, Zhang, Zou, & Han, 2015). In addition, because patients with advanced NSCLC are not only symptomatic, but often older with comorbidities that affect their quality of life, efficacious treatments with good tolerability profiles continue to be needed (Grønberg et al., 2010; NCI, 2015a).

## RATIONALE FOR USE OF EGFR TKIS

Due to the increasing need for effective and well-tolerated treatments, research has focused on identifying biomarkers to predict clinical benefit in specific subpopulations of patients and developing treatments that target those mutations. One of the first biomarkers to result in a pharmacotherapeutic agent with clinical utility was the epidermal growth factor receptor (EGFR).

**Figure 1:**
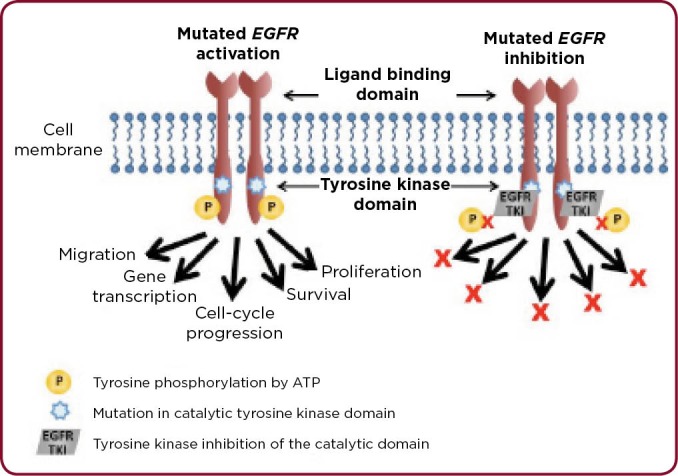
Mechanism of tumorigenesis by mutated EGFR and of inhibition by EGFR TKIs. ATP = adenosine triphosphate; EGFR TKI = epidermal growth factor receptor tyrosine kinase inhibitor.

EGFR (HER1, ERBB1) is a transmembrane protein that belongs to the HER/ERBB family of receptor tyrosine kinases (Bronte et al., 2014). In normal tissue, when a ligand binds to the extracellular domain of EGFR, it activates the tyrosine kinase domain, stimulating signaling pathways that regulate intracellular processes, such as proliferation, invasion, cellular repair, protection from injury, and cell survival (Arteaga, 2002; Bronte et al., 2011). In the setting of cancer, activating mutations in the tyrosine kinase domain result in the overstimulation of these signaling pathways to drive malignant transformation (Figure 1; Lynch et al., 2004; Sordella, Bell, Haber, & Settleman, 2004).

Clinically significant *EGFR* mutations occur in 30% to 50% of Asian populations and 7% to 14% of Western populations (Shigematsu et al., 2005; Sekine, Yamamoto, Nishio, & Saijo, 2008; Sequist et al., 2011; Rosell et al., 2012; Douillard et al., 2014; National Comprehensive Cancer Network [NCCN], 2016). In addition, a distinct difference in the prevalence of these mutations is seen among smokers (10%–20%) vs. nonsmokers (40%–60%; Shigematsu et al., 2005; Zhang et al., 2015).

Four agents that target the tyrosine kinase domain of EGFR are currently available in the United States: gefitinib (Iressa), erlotinib (Tarceva), afatinib (Gilotrif), and osimertinib (Tagrisso), which specifically targets the T790M mutation of EGFR. Based on the success of a number of pivotal trials in selected *EGFR* mutation–positive patients (Mok et al., 2009; Maemondo et al., 2010; Mitsudomi et al., 2010; Zhou et al., 2011; Rosell et al., 2012; Sequist et al., 2013; Wu et al., 2014), gefitinib, erlotinib, and afatinib are now recommended by the NCCN for first-line treatment for patients with advanced or metastatic *EGFR* mutation–positive (exon 19 deletions or exon 21 [L858R] substitution mutations) NSCLC (Lindeman et al., 2013; NCCN, 2016; AstraZeneca, 2015a; Genentech, 2015; Boehringer Ingelheim Pharmaceuticals, 2014). Osimertinib is recommended for the treatment of patients with metastatic NSCLC who progressed on or after EGFR TKI therapy and who exhibit a specific T790M mutation, as detected by a U.S. Food and Drug Administration (FDA)-approved test (NCCN, 2016; AstraZeneca, 2015b).

## ADVERSE EFFECTS ASSOCIATED WITH EGFR TKIS

In general, the available EGFR TKIs are well tolerated compared with traditional chemotherapy, but a few adverse effects are commonly seen in clinical use and are the focus of this review (Fiala et al., 2013; Burotto, Manasanch, Wilkerson, & Fojo, 2015; Haspinger et al., 2015). It should be noted that because osimertinib is relatively new (approved by the FDA in November 2015 in the United States), much of the information presented here is based on the experience with the three EGFR TKIs approved for first-line use.

Additionally, the adverse event experience with the first-line EGFR TKI agents is derived mainly from studies that enrolled treatment-naive patients, whereas the data related to osimertinib are from patient populations pretreated with one of the first-line EGFR TKI agents. These agents are all orally active but have different structures, molecular weights, and affinities for EGFR, which may explain their different toxicity profiles, as presented in Table 1 (Bronte et al., 2014; Modjtahedi, Cho, Michel, & Solca, 2014). Fatal events associated with EGFR TKI therapy are rare but have been reported in relation to lung or liver toxicity (Maemondo et al., 2010; Schacher-Kaufmann & Pless, 2010; Ren et al., 2012; Yang et al., 2012).

**Table 1:**
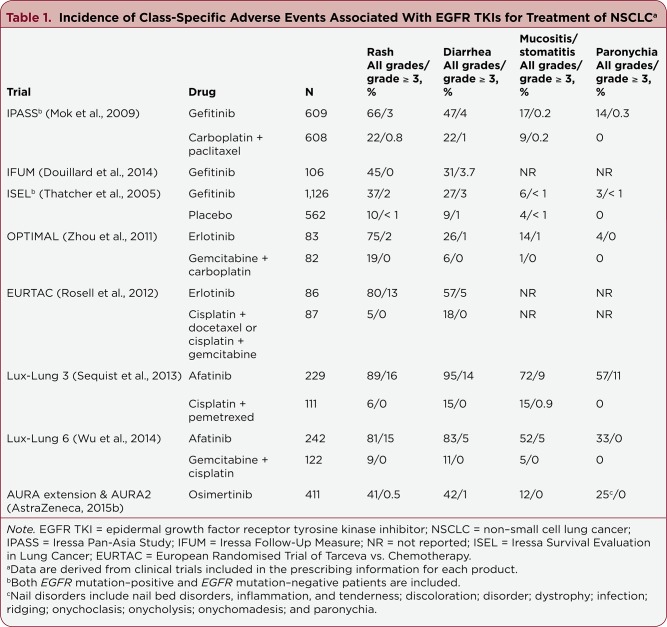
Incidence of Class-Specific Adverse Events Associated With EGFR TKIs for Treatment of NSCLCa

Here is an overview of the most common adverse events (AEs) and those of special interest associated with EGFR TKIs. It should be noted that incidence rates of the AEs listed and discussed here are derived from individual clinical trials, and comparisons among these EGFR TKI therapies are complicated by different patient populations and trial designs.

**Rash**

*Pathogenesis*: Dermatologic AEs are not unexpected, as EGFR is ubiquitously present in the skin (Galimont-Collen, Vos, Lavrijsen, Ouwerkerk, & Gelderblom, 2007). Normally found in epidermal and follicular keratinocytes, EGFR is the basal layer of the epidermis, outer root sheath of the hair follicle, sebaceous and eccrine epithelium, dendritic antigen–presenting cells, and connective tissue cells, where it plays a number of important roles. These roles include control of differentiation; protection from damage, such as that caused by ultraviolet radiation; inhibition of inflammation; and acceleration of wound healing (Mitchell, Perez-Soler, Van Cutsem, & Lacouture, 2007). Although the precise pathogenesis of dermatologic toxicity with EGFR TKIs is unclear, it is thought to be related to inflammatory processes resulting from interference by EGFR TKIs in the follicular and interfollicular epidermal growth signaling pathways, leading to changes in keratinocyte proliferation, differentiation, migration, and attachment (Woodworth et al., 2005; Mitchell et al., 2007; Lacouture, 2006).

*Clinical Presentation*: Rash associated with EGFR TKI use is seen in 37% to 66% of patients treated with gefitinib, 75% to 80% of patients treated with erlotinib, 81% to 89% of patients treated with afatinib, and 41% of patients treated with osimertinib (Table 1), and a recent meta-analysis also demonstrated a greater incidence of rash with afatinib than with the other two agents used as first-line therapy (Mok et al., 2009; Rosell et al., 2012; Sequist et al., 2013; Burotto et al., 2015; AstraZeneca, 2015a).

The rash associated with EGFR TKI use presents as the sudden onset of a papulopustular eruption; it is distinct from acne vulgaris in that it is associated with the characteristic papules and pustules, but there is a distinct absence of comedones (Eaby-Sandy, Grande, & Viale, 2012). The rash associated with EGFR TKI use generally involves the face, scalp, neck, upper chest, and back. In addition, diffuse erythema and telangiectasias can occur (Galimont-Collen et al., 2007). In rare cases, pustules congregate into pustular lakes with hard, yellow, adherent crusts (Galimont-Collen et al., 2007).

The rash may initially present in the first week as sensory disturbances, erythema, and edema, followed by papulopustular eruptions in the second week and crusting in the fourth week (Lacouture & Melosky, 2007; Melosky & Hirsh, 2014). In most cases, these effects are temporary and may diminish in intensity with continued exposure to EGFR TKI treatment. As the rash subsides, a background of erythema and dry skin may be apparent in those areas previously affected by the papulopustular eruption, and postinflammatory hyperpigmentation and telangiectasias may also occur (Galimont-Collen et al., 2007; Lacouture & Melosky, 2007). No relation between the occurrence of rash and a history of oily skin, acne, rosacea, or skin type has been observed (Galimont-Collen et al., 2007).

**Figure 2:**
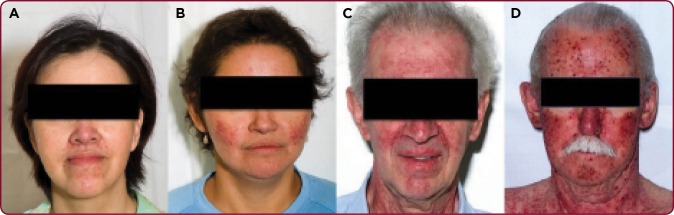
Acneiform rash induced by EGFR TKIs. (A) Grade 1, gefitinib; (B) grade 2, erlotinib; (C) grade 3, erlotinib; (D) grade 4, erlotinib. EGFR TKI = epidermal growth factor receptor tyrosine kinase inhibitor. Reproduced with permission from Melosky et al. (2015). Copyright 2015 by Multimed Inc.

*Severity*: The progression of rash from NCI Common Terminology Criteria for Adverse Events (CTCAE) grades 1 through 4 is shown in Figure 2. In the majority of cases, the rash related to EGFR TKI use is mild to moderate (grade 1 or 2), but severe (grade 3 or 4) rash is seen in about 3% of patients treated with gefitinib, 13% of patients treated with erlotinib, 16% of patients treated with afatinib, and 0.5% of patients treated with osimertinib (Table 1; Thatcher et al., 2005; Perez-Soler, 2006; Mok et al., 2009; Rosell et al., 2012; Sequist et al., 2013; Douillard et al., 2014; AstraZeneca, 2015a, 2015b).

Even mild rash has been shown to affect patients’ quality of life (Joshi et al., 2010). Patients have also reported discomfort serious enough to disrupt sleep and impair their ability to work or enjoy activities of daily living and hobbies (Wagner & Lacouture, 2007). Some patients are so self-conscious about their rash or skin changes they do not want to be seen in public.

*Management*: Management of dermatologic AEs involve both preemptive interventions and treatment after symptoms occur. Before EGFR TKI treatment, patients should be advised to moisturize their entire body at least twice daily, using a thick, alcohol-free emollient. Sun exposure should be minimized, and if unavoidable, a broad-spectrum physical sunscreen with a sun protection factor of at least 15 should be used in addition to wearing protective clothing, including a wide-brimmed hat. Zinc oxide or titanium dioxide–containing sunscreens are preferred because of their broad-spectrum protection and noncomedogenic properties.

The Skin Toxicity Evaluation Protocol With Panitumumab (STEPP) trial compared preemptive skin treatment (e.g., skin moisturizers, sunscreen, topical steroid, doxycycline) with reactive treatment after development of rash (any treatment deemed necessary by the investigator) in patients receiving panitumumab (Vectibix), an intravenous anti-EGFR monoclonal antibody used for colorectal cancer (Lacouture et al., 2010). The preemptive therapy group saw a > 50% reduction in the incidence of grade 2 or higher skin toxicity compared with the reactive group (Lacouture et al., 2010).

Although current guidelines do not recommend prophylactic drug regimens for rash, a number of studies have investigated different agents, but only oral minocycline showed any utility in patients with NSCLC treated with erlotinib in the second- and third-line settings (Scope et al., 2007; Jatoi et al., 2011; Melosky et al., 2016). Neither oral tetracycline nor topical tazarotene (Tazorac, Avage) had an effect on the occurrence or severity of rash induced by anti-EGFR treatment (Scope et al., 2007; Jatoi et al., 2011).

Patients should be reminded that although the rash associated with EGFR TKI therapy may have a similar appearance to acne, it should not be similarly treated, as anti-acne medications tend to be drying and may cause or exacerbate pruritus and irritation (Hirsh, 2011). In addition, the use of products that can dry the skin, such as soaps and alcohol-based or perfumed products, should be avoided, and shower time should be limited and consist of lukewarm rather than hot water (Hirsh, 2011).

It is recommended that patients be assessed weekly for any signs of rash during the first 6 weeks of treatment with an EGFR TKI and every 6 to 8 weeks thereafter (Hirsh, 2011). Patients, caregivers, and clinicians should all be educated to recognize the initial signs of rash, because early intervention may minimize the worsening of symptoms.

If signs or symptoms of rash manifest, the appropriate intervention protocol will depend on the severity/grade. For patients who experience mild rash (grade 1), no intervention may be necessary, but topical hydrocortisone 2.5% and/or clindamycin 1% can be considered (Brown, 2015). Moderate rash (grade 2) may be treated with hydrocortisone 2.5% plus either oral doxycycline (100 mg twice daily) or minocycline (100 mg twice daily; Brown, 2015). For patients experiencing more severe rash (grade 3/4), in addition to the treatments recommended for moderate cases, a methylprednisolone dose pack may be considered (Hirsh, 2011). It should be noted that the occurrence and/or severity of rash may fluctuate during the course of EGFR TKI therapy, and as such, treatment with creams and antibiotics may need to be repeated several times. Because of the pervasiveness of symptoms on overall well-being and the complexity of management, referral to a dermatology specialist is recommended for patients who experience severe (grade 3/4) skin-related toxicities. When the rash is treated promptly, progression to a grade 3 rash can often be avoided.

If symptoms do not improve or stabilize within 2 to 4 weeks or are grade ≥ 3 at the onset, treatment interruption or reduction of the dose of the EGFR TKI may be required (Hirsh, 2011; Melosky & Hirsh, 2014). Therapy with an EGFR TKI should be stopped for 10 to 14 days for a grade 3/4 rash until the rash improves to ≤ grade 2, at which time treatment can be reinitiated. For gefitinib, reinstatement of the full dose is recommended if a grade 3/4 rash improves to grade 1 or resolves (AstraZeneca, 2015a). The dose of erlotinib should be titrated down in 50-mg increments to a minimum of 50 mg and then increased in 50-mg increments back to the original dose as tolerated (Genentech, 2015). Afatinib should be restarted at a dose that is 10 mg/day less than the original dose (Boehringer Ingelheim Pharmaceuticals, 2014). Osimertinib should be withheld for up to 3 weeks for grade 3 adverse events and then restarted at a full- (80 mg) or half- (40 mg) dose daily when symptoms improved to grade 0 to 2 (AstraZeneca, 2015b). If the rash does not sufficiently resolve with interruption of EGFR TKI therapy, treatment should be permanently stopped.

**Figure 3:**
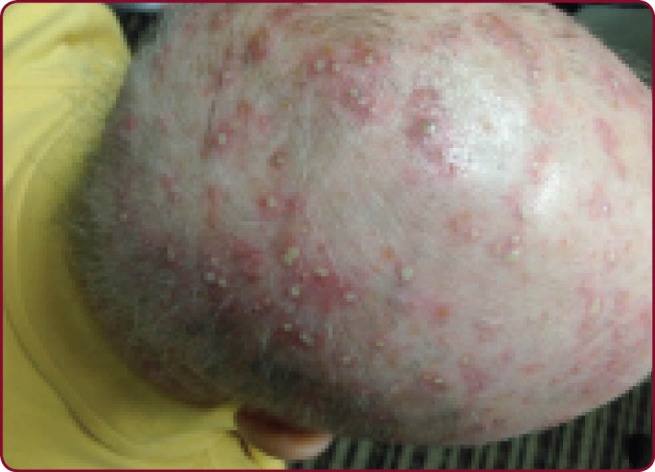
Rash of the scalp induced by an EGFR TKI. EGFR TKI = epidermal growth factor receptor tyrosine kinase inhibitor. Photo courtesy of Wendy Vogel.

Rash affecting the scalp (Figure 3), as opposed to other parts of the body, may be more difficult to treat. Patients may prefer a gel formulation of clindamycin or a steroid rather than a cream, as creams can be unpleasant to use on the hairline. In addition, scalp changes can be treated with a formulation of topical clindamycin 2% plus triamcinolone acetonide 0.1% in equal parts of propylene glycol and water (Melosky & Hirsh, 2014).

**Diarrhea**

*Pathogenesis*: As in the skin, EGFR is expressed in abundance in the mucosa of the gastrointestinal (GI) tract and plays an important role in normal gut development and maintenance of epithelial continuity (Goodlad & Wright, 1995). The EGFR TKIs are thought to disrupt these functions, thereby leading to diarrhea.

*Clinical Presentation*: Diarrhea associated with EGFR TKI use occurs in 27% to 47% of patients treated with gefitinib, 26% to 57% of patients treated with erlotinib, 83% to 95% of patients treated with afatinib, and 42% of patients treated with osimertinib (Table 1; Thatcher et al., 2005; Mok et al., 2009; Rosell et al., 2012; Sequist et al., 2013; Douillard et al., 2014; AstraZeneca, 2015a, 2015b). A relatively recent meta-analysis noted a higher rate of diarrhea with afatinib (Burotto et al., 2015).

Diarrhea usually occurs during the first 4 weeks of initiation of gefitinib and erlotinib and within the first 7 days of initiation of afatinib (Hirsh, Blais, Burkes, Verma, & Croitoru, 2014). Patients should be monitored weekly during this period. Diarrhea may cause physical discomfort, fatigue, and sleep disturbances, as well as affect social functioning. Nutritional deficits may occur secondary to diarrhea, and severe diarrhea can lead to dehydration, electrolyte imbalance, and renal insufficiency.

*Management*: Treatment choices for diarrhea are based on its cause, severity/grade, and clinical presentation (e.g., symptom duration and stool characteristics), plus any coexisting symptoms. Before initiating a management plan for diarrhea, it is essential to rule out other possible causes of diarrhea, which can include medications (e.g., laxatives, stool softeners, antibiotics, antacids); dietary factors, such as excess consumption of fiber, dairy products, or greasy foods; comorbid infections (e.g., intestinal obstruction, fecal impaction, surgery); or radiation toxicity (Melosky & Hirsh 2014).

The diagnostic workup may include a complete blood cell count to rule out neutropenia or infection, a chemistry panel to assess renal function and electrolyte abnormalities, and stool culture or *Clostridium difficile* toxin screen to test for bacterial pathogens. Diagnostic scans or endoscopy may be indicated to rule out conditions such as bowel obstruction or perforation. A thorough history is important to characterize the type of diarrhea, timing of onset, duration, severity, associated symptoms, and any contributing or alleviating factors.

Nonpharmacotherapeutic interventions for diarrhea include dietary changes, fluid intake, and probiotics (NCI, 2015b). The BRAT diet (bananas, rice, applesauce, toast) can be prescribed for short-term management. Foods that may worsen symptoms should be avoided. Preemptive dietary changes before the occurrence of diarrhea are not recommended. Patients are encouraged to consume about 3 to 4 liters of liquids to prevent dehydration, including fluids with sugar and salt to avoid electrolyte imbalances. Caffeinated and alcoholic beverages should be avoided. Although it is not recommended to treat diarrhea preemptively before its onset due to the risk of constipation, patients should be educated to begin over-the-counter loperamide at the start of symptoms.

Patients with grade 1 diarrhea can be started on 4 mg of loperamide at symptom initiation and then 2 mg after each loose stool, for a maximum of 20 mg daily (Hirsh et al., 2014). This regimen may be continued until there have been no episodes of diarrhea for 12 hours. If the diarrhea does not resolve or becomes moderate (grade 2), the same regimen should be followed. Patients experiencing grade 3/4 diarrhea may require hospitalization, with continuation of loperamide plus aggressive intravenous fluid replacement. For patients who are neutropenic, antibiotics may be administered prophylactically. If loperamide is contraindicated or ineffective, diphenoxylate-atropine (5 mg [2 tablets] 4 times daily for a maximum of 20 mg daily) or codeine (30 mg every 4 hours) can be substituted; either treatment can be added if symptoms are not controlled.

As with rash, for patients with diarrhea, all efforts should be made to maintain treatment with the EGFR TKI, but if a temporary discontinuation is necessary, treatment should be reinstated using the same protocols previously mentioned. If diarrhea does not resolve to grade ≤ 1 within 14 days of withholding treatment and providing supportive care, permanent discontinuation of EGFR TKI therapy must be considered (Hirsh et al., 2014).

## OTHER ADVERSE EVENTS ASSOCIATED WITH EGFR TKIS

**Mucositis/Stomatitis**

Mucositis and stomatitis can be troublesome for patients receiving EGFR TKI therapy. These inflammatory conditions of oral tissue encompass not only the mucosa but also dentition and the surrounding gums. The rates of mucositis/stomatitis vary with the current EGFR TKIs, with incidence rates of 6% to 17% for gefitinib, 14% for erlotinib, 52% to 72% for afatinib, and 12% for osimertinib (Table 1; Thatcher et al., 2005; Mok et al., 2009; Sequist et al., 2013; Douillard et al., 2014; Genentech, 2015; AstraZeneca, 2015a, 2015b).

Symptoms range from mild tingling in the mouth or tongue to painful ulcers and cracks on the sides of the mouth, which make eating and drinking difficult. In addition, ulcerations in the oral mucosa can provide a point of entry for microorganisms, potentially leading to systemic infection (Bensinger et al., 2008). Referral to a dentist should be considered to prevent these serious sequelae. Baseline oral assessments are documented, and patients are educated about oral hygiene practices, such as frequent brushing with soft bristles, flossing, and rinsing with saline or sodium bicarbonate.

Xerostomia may improve with mouth rinses, which stimulate salivary gland function; however, alcohol-containing mouthwashes should be avoided, as they can cause further irritation to the oral mucosa. Angular cheilitis can be treated by the application of barrier ointments or hydrocortisone cream; however, evaluation for an underlying infectious or fungal etiology should be considered if symptoms do not improve. Grade 1 or 2 mucositis/stomatitis can be treated with triamcinolone acetonide dental paste applied (by dabbing not rubbing) two or three times daily, and erythromycin (250–350 mg) can be added if symptoms are more severe (grade 2; Melosky & Hirsh, 2014). For grade 3 mucositis/stomatitis, clobetasol ointment may be substituted for the triamcinolone acetonide, and the dose of erythromycin can be increased to 500 mg. Therapy with an EGFR TKI should be suspended until improvement to grade ≤ 2 is observed, at which time treatment may resume following the same guidelines previously discussed (Melosky & Hirsh, 2014).

**Paronychia**

**Figure 4:**
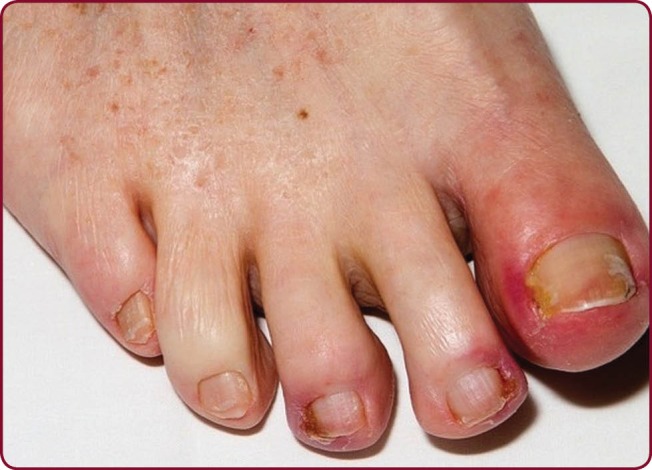
Grade 3 paronychia of the toes induced by erlotinib. Reproduced with permission from Melosky et al. (2015). Copyright 2015 by Multimed Inc.

Paronychia consists of inflammation, soreness, or infection around the nail beds of the fingers and toes (Figure 4; Melosky & Hirsh, 2014). Although rarely seen with erlotinib (~4%) and gefitinib (3%–14%), paronychia is more common with afatinib (33%–57%) and osimertinib (~25%; Table 1; Thatcher et al., 2005; Mok et al., 2009; Rosell et al., 2012; Sequist et al., 2013; Douillard et al., 2014; AstraZeneca, 2015a, 2015b). It can be difficult to manage and can become more of a concern with longer treatment durations (Eaby-Sandy, 2010; Melosky & Hirsh, 2014). Most instances of paronychia related to EGFR TKI therapy are mild, but in clinical trials of afatinib, treatment-related paronychia led to dose reductions in 14% of patients (Melosky & Hirsh, 2014; Boehringer Ingelheim Pharmaceuticals, 2014).

A baseline assessment of fingernails, toenails, and the surrounding tissue should be documented. Daily activities of each patient should be reviewed, noting factors that might increase the likelihood or severity of paronychia, such as work-related exposure to skin irritants like chemicals or frequent hand washing. Patients should be educated about measures of prevention, such as avoiding topical irritants; good hand washing; keeping nails trimmed; avoiding extreme temperatures, friction, or other injury; and using emollient creams or ointments around the nails. Patients may also benefit from wearing gloves while doing housework to minimize exposure to cleaning agents and to avoid impact injury.

Recommended interventions for grade 1 paronychia (mild and localized) include topical antibiotics and antiseptics, such as clindamycin 1%, erythromycin 1%, tetracycline 1%, chloramphenicol 1%, and iodine ointment (Melosky & Hirsh, 2014). Soaking nails with Epsom salts, diluted Betadine, bleach, or vinegar may also help (Eaby-Sandy, 2010; Melosky & Hirsh, 2014). Weekly application of silver nitrate may be additionally recommended for grade 2 paronychia (moderate; Brown, 2015). If patients experience grade 3 paronychia (severe), the EGFR TKI should be discontinued until symptoms resolve (Brown, 2015). The use of a liquid bandage may reduce pain and the risk of infection from fissures and/or splinter. Oral antibiotics may be indicated, with culturing of suspected infections, and empiric antibiotic therapy is recommended (Eaby-Sandy, 2010). Referral to a dermatologist is recommended for patients with paronychia that affects well-being or is unresponsive to treatment.

**Ocular-Related Toxicity**

Ocular-related adverse events most likely arise because EGFR is expressed on corneal, limbal, and conjunctival epithelium, and inhibition of EGFR affects the epithelial cell proliferation and stratification necessary for corneal wound repair (Saint-Jean et al., 2012). The occurrence of ocular-related AEs with EGFR TKIs varies with the agent. With gefitinib, about 7% of patients have experienced conjunctivitis/blepharitis/dry eye, and about 0.1% have reported keratitis (AstraZeneca, 2015a). Erlotinib has been associated with decreased tear production, abnormal eyelash growth, keratoconjunctivitis sicca, and keratitis, potentially leading to corneal perforation and ulceration, in about 18% of patients in lung cancer studies (Genentech, 2015). About 11% of patients treated with afatinib experienced conjunctivitis (Boehringer Ingelheim Pharmaceuticals, 2014). About 18% of patients treated with osimertinib experienced eye disorders, defined as dry eye, blurred vision, keratitis, cataract, eye irritation, blepharitis, eye pain, increase in lacrimation, and vitreous floaters; other ocular toxicities occurred in < 1% of patients (AstraZeneca, 2015b).

For external disorders, patients may benefit from gently washing the eyelashes with diluted baby shampoo. Mild dry eye may be managed with the use of natural tear eye drops. Patients who develop ocular complications should be referred to an ophthalmologist, and it may be necessary to discontinue EGFR TKI therapy until symptoms improve (Saint-Jean et al., 2012; Boehringer Ingelheim Pharmaceuticals, 2014; Genentech 2015; AstraZeneca, 2015a, 2015b).

**Interstitial Lung Disease**

Although rare, interstitial lung disease (ILD) and ILD-like events are important because some can be fatal (Qi, Sun, Shen, & Yao, 2015). The etiology of ILD associated with EGFR TKI therapy is not fully understood but is thought to be related to inhibition of EGFR and its family members, which are upregulated early in the response to acute lung injury and contribute to the repair of pulmonary damage (Qi et al., 2015). Interstitial lung disease is also known to be associated with the natural history of NSCLC and the many diverse classes of compounds, including conventional chemotherapy (Camus, 2004).

The overall incidence of ILD (all grades) with gefitinib was 1.3% (AstraZeneca, 2015a). The incidence rate with erlotinib was similar, at 1.1% (Genentech, 2015). Approximately 1.5% of patients who received afatinib experienced ILD or ILD-like AEs (Boehringer Ingelheim Pharmaceuticals, 2014), and 3.3% of patients treated with osimertinib experienced ILD/pneumonitis (AstraZeneca, 2015b). The incidence of ILD appears to be higher in never-smokers and in those of East Asian descent (Boehringer Ingelheim Pharmaceuticals, 2014; Qi et al., 2015).

Risk factors for ILD include a history of current smoking, preexisting lung disease, reduced lung volume, cardiovascular disease, older age, and poorer performance status. Patients should be screened at each visit for signs of ILD, which include acute onset of dyspnea, possibly associated with cough or low-grade fever. Symptoms may become exacerbated enough within a short period to require hospitalization. It is recommended that EGFR TKI therapy be permanently discontinued in patients who develop ILD (AstraZeneca, 2015a, 2015b; Qi et al., 2015).

## ADDITIONAL CONSIDERATIONS

**Rash and Outcomes**

It is thought that because the efficacy of the three first-line EGFR TKI agents is relatively similar, but the incidence of rash varies, rash may be an indicator of drug exposure rather than efficacy and may be related to drug dose (Rukazenkov et al., 2009; Liu et al., 2013). Erlotinib is typically prescribed at its reported maximum tolerated dose, and gefitinib is administered at only about one-third of its maximum tolerated dose; therefore, the toxicity threshold of erlotinib may be similar to the concentration necessary for therapeutic effect, whereas there is a larger therapeutic window with gefitinib (Rukazenkov et al., 2009; Peters, Zimmermann, & Adjei, 2014). In addition, gefitinib selectively accumulates in tumor tissue, limiting its exposure in the circulation and thereby potentially lowering the risk of rash (Rukazenkov et al., 2009; Haura, Sommers, Song, Chiappori, & Becker, 2010). It is also postulated that the development of rash may be related to the immune status of the patient, possibly reflecting a healthier immune system and therefore a better prognosis (Perez-Soler & Van Cutsem, 2007).

**Bioavailability**

The bioavailability of erlotinib and afatinib increases with food intake, which may increase toxicity; therefore, patients are instructed to take these two agents on an empty stomach (Peters et al., 2014; Boehringer Ingelheim Pharmaceuticals, 2014; Genentech, 2015). Food does not affect the bioavailability of gefitinib or osimertinib, and they can both be dissolved in a glass of water or administered through a nasogastric tube if patients have difficulty swallowing solids (Peters et al., 2014; AstraZeneca 2015a, 2015b). Histamine (H2)-receptor antagonists and proton pump inhibitors, both commonly used medications by patients with cancer, have been shown to decrease the absorption and bioavailability of gefitinib and erlotinib, which may reduce the occurrence of AEs but may also reduce their efficacy (Peters et al., 2014; AstraZeneca, 2015a; Genentech, 2015). Exposure to osimertinib was not affected by the concurrent administration of the proton pump inhibitor omeprazole (AstraZeneca, 2015b). Afatinib is highly soluble over a range of physiologic pH levels and therefore is not expected to be affected by acid-reducing drugs (Peters et al., 2014). The package inserts of each agent should be consulted for information about administration of the EGFR TKIs with agents that may affect bioavailability.

**Drug-Drug Interactions**

Gefitinib is metabolized primarily in the liver by cytochrome P450 (CYP) 3A4 and to a lesser extent by CYP2D6 and CYP3A5, whereas erlotinib is metabolized by CYP3A4/3A5 and to a lesser extent by the CYP1A1/1A2 isoenzymes (Peters et al., 2014). Afatinib is metabolized by P-glycoprotein transporters in the liver. The main metabolic pathways of osimertinib are oxidation by CYP3A and dealkylation (AstraZeneca, 2015b). Therefore, coadministration with drugs that inhibit these pathways may increase exposure and thereby AEs. Drugs shown to increase exposure of gefitinib and erlotinib include the azole antifungals, protease inhibitors, clarithromycin, telithromycin, ciprofloxacin, and fluvoxamine. Strong CYP3A inhibitors have also been shown to increase osimertinib plasma concentrations (AstraZeneca, 2015b). Afatinib exposure may be increased by concomitant administration of ritonavir, cyclosporin A, ketoconazole, itraconazole, erythromycin, verapamil, quinidine, tacrolimus, nelfinavir, saquinavir, and amiodarone (Peters et al., 2014).

## CONCLUSIONS

Therapy with EGFR TKIs does not cause many of the common toxicities seen with chemotherapy and have proved to be effective options for patients with NSCLC who harbor *EGFR* mutations. The unique mechanism of action of the EGFR TKIs allows for a better tolerability profile, but as a result of these agents targeting EGFR, rash and diarrhea are commonly encountered.

Before beginning any treatment regimen for cancer, patients should be educated on any possible AEs, and nurses and advanced practitioners play an important role in providing this information. Patients should also be instructed on various preventive measures prior to initiating EGFR TKI therapy (Table 2). Patients should be educated to recognize the early signs and symptoms of AEs, as well as be encouraged to report any adverse reactions, as early intervention is critical for optimal management. In addition, because rash and diarrhea are often associated with infectious etiologies, it is important to reassure patients that their conditions are due to the adverse effects of the drugs and therefore not transmittable to others.

**Table 2:**
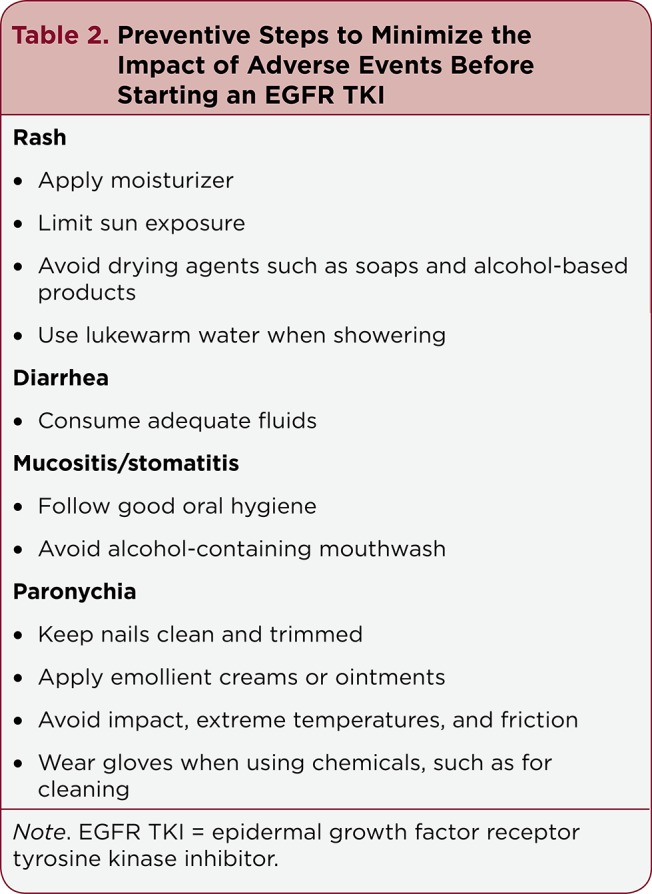
Preventive Steps to Minimize the Impact of Adverse Events Before Starting an EGFR TKI

Because EGFR TKIs are administered orally, adherence can be suboptimal, especially if undue toxicity occurs. Therefore, practitioners should emphasize the importance of adhering to treatment and reporting AEs; assessments should occur at every visit and should be documented. Prompt management of toxicities, temporary treatment discontinuation, and appropriate dose reduction should help keep patients on effective therapy, which is essential to optimal outcomes. Prompt management of all side effects related to EGFR TKI use is essential to maintaining patient compliance, so patients can realize the full clinical benefit of their prescribed treatments.

**Acknowledgments**

The authors wish to thank Meredith Rogers, MS, CMPP, of The Lockwood Group, for providing writing and editorial assistance funded by AstraZeneca. The authors did not receive any honoraria for this publication.
